# Deep Learning With Sampling in Colon Cancer Histology

**DOI:** 10.3389/fbioe.2019.00052

**Published:** 2019-03-27

**Authors:** Mary Shapcott, Katherine J. Hewitt, Nasir Rajpoot

**Affiliations:** ^1^Department of Computer Science, University of Warwick, Coventry, United Kingdom; ^2^Cellular Pathology Department, University Hospital of Coventry and Warwickshire, Coventry, United Kingdom

**Keywords:** sampling, histopathology, TCGA, morphology, colon cancer, deep learning

## Abstract

This study applied a deep-learning cell identification algorithm to diagnostic images from the colon cancer repository at The Cancer Genome Atlas (TCGA). Within-image sampling improved performance without loss of accuracy. The features thus derived were associated with various clinical variables including metastasis, residual tumor, venous invasion, and lymphatic invasion. The deep-learning algorithm was trained using images from a locally available data set, then applied to the TCGA images by tiling them, and identifying cells in each patch defined by the tiling. In this application the average number of patches containing tissue in an image was ~900. Processing a random sample of patches greatly reduced computation costs. The cell identification algorithm was applied directly to each sampled patch, resulting in a list of cells. Each cell was labeled with its location and classification (“epithelial,” “inflammatory,” “fibroblast,” or “other”). The number of cells of a given type in the patch was calculated, resulting in a patch profile containing four features. A morphological profile that applied to the entire image was obtained by averaging profiles over all patches. Two sampling policies were examined. The first policy was random sampling which samples patches with uniform weighting. The second policy was systematic random sampling which takes spatial dependencies into account. Compared with the processing of complete whole slide images there was a seven-fold improvement in performance when systematic random spatial sampling was used to select 100 tiles from the whole-slide image for processing, with very little loss of accuracy (~4% on average). We found links between the predicted features and clinical variables in the TCGA colon cancer data set. Several significant associations were found: increased fibroblast numbers were associated with the presence of metastasis, venous invasion, lymphatic invasion and residual tumor while decreased numbers of inflammatory cells were associated with mucinous carcinomas. Regarding the four different types of cell, deep learning has generated morphological features that are indicators of cell density. The features are related to cellularity, the numbers, degree, or quality of cells present in a tumor. Cellularity has been reported to be related to patient survival and other diagnostic and prognostic indicators, indicating that the features calculated here may be of general usefulness.

## Introduction

Histopathology, the microscopic examination of diseased tissue is central to the diagnosis and treatment of cancer. Recent developments in digital microscopy have enabled the extraction of useful information from whole-slide images (WSIs) of cancer tissue using deep learning algorithms that are based on convolutional neural networks (Janowczyk and Madabhushi, 2016). Ideally, deep learning applications can replace tasks carried out in manual pathology, identifying key features that are easily interpretable and that have prognostic power.

Most deep learning algorithms are trained with relatively small images. To apply a trained algorithm to a whole-slide image a straightforward approach is to tile the WSI with small patches and apply the deep-learning algorithm to each patch independently. The per-patch results may be averaged over the WSI to generate a collection of features which characterize the spatial characteristics of the WSI, a *morphological profile*.

However, such an approach is computationally costly: on average, each WSI in the data set used in this study contained about 900 patches that had significant amounts of tissue. Computational costs can be reduced by sampling a limited number of patches, applying the algorithm to each, then averaging the per-patch features. In principle, if enough patches are sampled, processing costs can be reduced without significant loss of accuracy. The main aim of this study was to examine the behavior of sampling as applied to WSIs. In addition, we have showed how profiles generated by sampled patches have significant associations with clinical variables.

**Figure 1:**
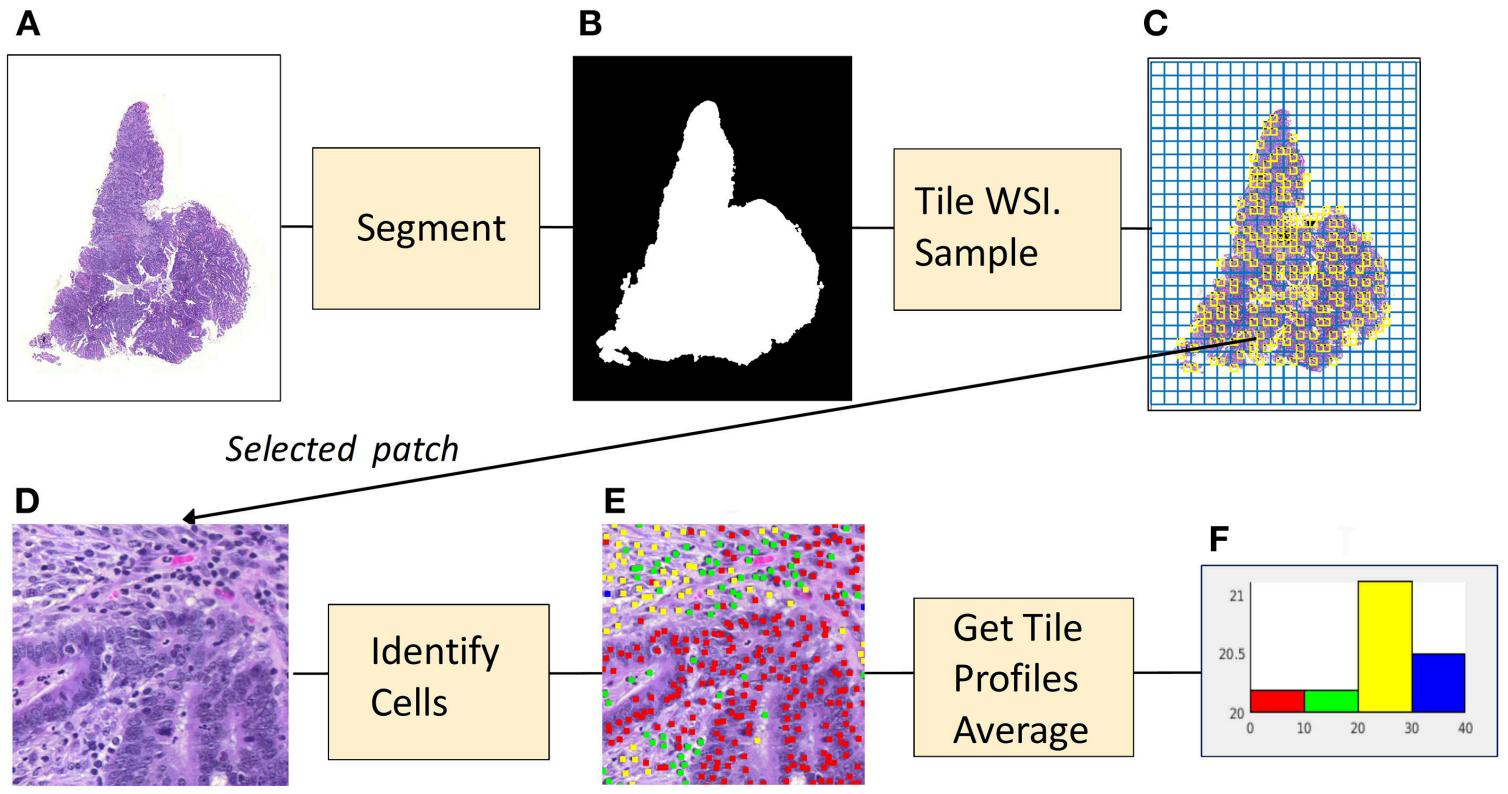
From image to profile: stages. **(A)** Histopathology Image. **(B)** Foreground Mask. **(C)** Tiling, with sampled patches. **(D)** Sampled patch. **(E)** Cells identified by algorithm. **(F)** Histogram of cell frequencies.

Figure 1 illustrates the stages used to create a morphological profile from a whole-slide image. The cell identification algorithm has been trained using images of fixed size and resolution. (In the experiments described here, the training image size was 500 × 500 pixels at a resolution of 20X, ~0.5 microns/pixel). The algorithm identifies cells (in practice cell nuclei) and classifies them as one of four types (epithelial cells, inflammatory cells, fibroblasts, and “other” cells).

In the first stage, that of segmentation, the whole slide image (1a) is separated into foreground and background regions, represented by a binary mask (1b). In the second stage the mask is divided into patches which are the same size and resolution as those used to train the algorithm. Each patch then is categorized as foreground or background, depending on the percentage of pixels assigned by the mask. Next, as shown in (1c) foreground patches in the grid of tiles are sampled. For each patch (1d) that has been sampled the cell identification algorithm locates and classifies cells (1e). The information concerning cell nuclei is summarized in a *tile profile*. The tile profiles are averaged, generating the profile for the whole-slide image. The profile is displayed as a histogram (1f) showing the frequencies of the four cell types: epithelial, inflammatory, fibroblasts and “other.” The features comprising the profile can be interpreted as measures of *cellularity*, the number of cells of a given type in the cancer tissue. Cellularity is described as:

“The degree, quality, or condition of cells that are present” (Farlex Partner Medical Dictionary, 2012).

Sampling of regions within an image is a standard procedure in manual pathology. Pathologists are accustomed to rapidly scanning tissue slides under the microscope and selecting interesting regions for intensive consideration. Kayser et al. (2009) discuss how an equivalent procedure can be carried out using digital pathology. They propose an implementation using three stages. In the first stage a set of regions in the image is generated by automated sampling, in the second stage an information measure is calculated for each region, and in the final stage the most informative regions are selected for intensive consideration by the pathologist. The authors argue that this hybrid approach can achieve viewing times that are comparable with those achieved in manual pathology.

Automated sampling within an image is used in *stereology*, originally the analysis of three-dimensional structures, using two-dimensional sections. In stereology various statistical procedures are used to extract significant structural information. A typical approach is to lay a regular grid over the image, and to sample the image using the grid. Stereology has been applied using digital pathology by Keller et al. (2013). The authors found that the use of their automated sampling algorithm was 50–90% more time efficient than conventional random sampling.

In another study sampling was employed in the analysis of cases of colon cancer where pathologists were asked to categorize the tissue type at 300 randomly selected points in a dense region of tissue (West et al., 2010). The study found that a low proportion of tumor cells was related to poor cancer-specific survival.

As for digital pathology, a description of the use of sampling in the detection of invasive breast cancer in histopathology images can be found in Cruz-Roa et al. (2018). A trained CNN classifier accepted patches of fixed size as input. The pathology image was tiled and in the first sampling step the resulting patches were randomly sampled. Each patch in the sample set was classified as homogeneous or heterogeneous. Regions of interest were those where the classification was uncertain. The regions surrounding tiles of uncertain classification were searched by sampling them systematically, using the gradient of the uncertainty map to guide the search.

In histopathology applications the choice of a sampling policy is affected by spatial dependency, whereby characteristics at neighboring locations tend to have similar values. Standard statistical sampling techniques that assume independence among observations do not take spatial dependency into account and are not always the most appropriate. Sampling policies that do take account of spatial dependencies have been developed in geospatial statistics (Delemelle, 2009) and of these systematic random sampling is a well-known technique (De Smith, 2018).

In the experiments described in this article a straightforward approach has been used: sampling of a set of patches, followed by cell identification, and profile generation. The two sampling policies that have been implemented are random sampling, and systematic random sampling.

Note that in some situations, non-random sampling, such as uniform spacing may be adequate. Uniform spacing gives good coverage of the WSI but will fail if there are periodicities in the image, or if there are relationships that depend on distance that should be estimated from the sample.

In the work described here, variants of two sampling policies have been implemented. In the basic form of *Random Sampling* (RS) a set of *N* points is selected from a W × H sized rectangle, using the uniform distribution over [W, H]. Random sampling is straightforward to implement but if spatial dependencies are present random sampling tends over-sample some areas and under-sample others.

*Systematic Random Sampling* (SRS) overcomes the unbalanced sampling problem of RS. The region surrounding the image is overlaid with a grid of identical tiles and a sample is taken from within each tile. SRS may be viewed as a combination of random and non-random sampling.

In *adaptive sampling*, information is derived from the samples already taken, and used to choose later samples. If elements of search are incorporated into the sampling process, then adaptive sampling may be appropriate. SRS and RS are non-adaptive sampling policies: all observations are made at once, according to the same rule.

This article reports on experiments with sampling polices, RS and SRS. Because there was no prior information to indicate that any specific feature in the morphological profile should be prioritized, we did not consider the use of adaptive sampling. This does not rule out the use of adaptive sampling in future applications, for example, when it is necessary to concentrate on features that are uncommon and when sampling should be directed toward areas with such features. For example, if a tissue sample consists mainly of normal cells, but we wish to analyze the features of abnormal cells, it might be advisable to search near points already sampled that were found to contain abnormal cells.

## Materials and Methods

### Cell Identification Algorithm

To enable the comparison of the two sampling policies in the calculation of WSI profiles a cell identification algorithm was trained, based on work described in Sirinukunwattana et al. (2016). The algorithm comprised two convolutional neural networks (CNNs) working in series. The first network was a detection network which located cells and passed the cell coordinates to the second network, a classification network which categorized each cell as epithelial, inflammatory, a fibroblast or as “other.” The algorithm was trained using a dataset augmented from that used in Sirinukunwattana et al. (2016). Training data was a set of RGB images of size [500 × 500] at 20X. The output of the cell identification algorithm was a cell map—a set of cell locations and cell types.

In equation (1) the model accepts an image *I*, and creates a cell-map consisting of *n*_*M*_ cell nuclei, located at points <*x,y*> each of which is labeled by a cell type *c*.

(1) $$\begin{array}{r} M\left(I\right)\;=\left\{n_{M},\;x_{i},y_{i},\;c_{i}:1\leq i\leq n_{M}\right\}\#\left(1\right) \end{array}$$

The image's morphological profile is a set of *J* features. Feature *f*_*j*_ is the number of cells of type *j* in the cell map:

(2) $$\begin{array}{r} f_{j}=\overset{n_{M}}{\underset{i}{\sum }}\left\{\left(c_{i}\right\}==j\right)\#\left(2\right) \end{array}$$

**Figure 2:**
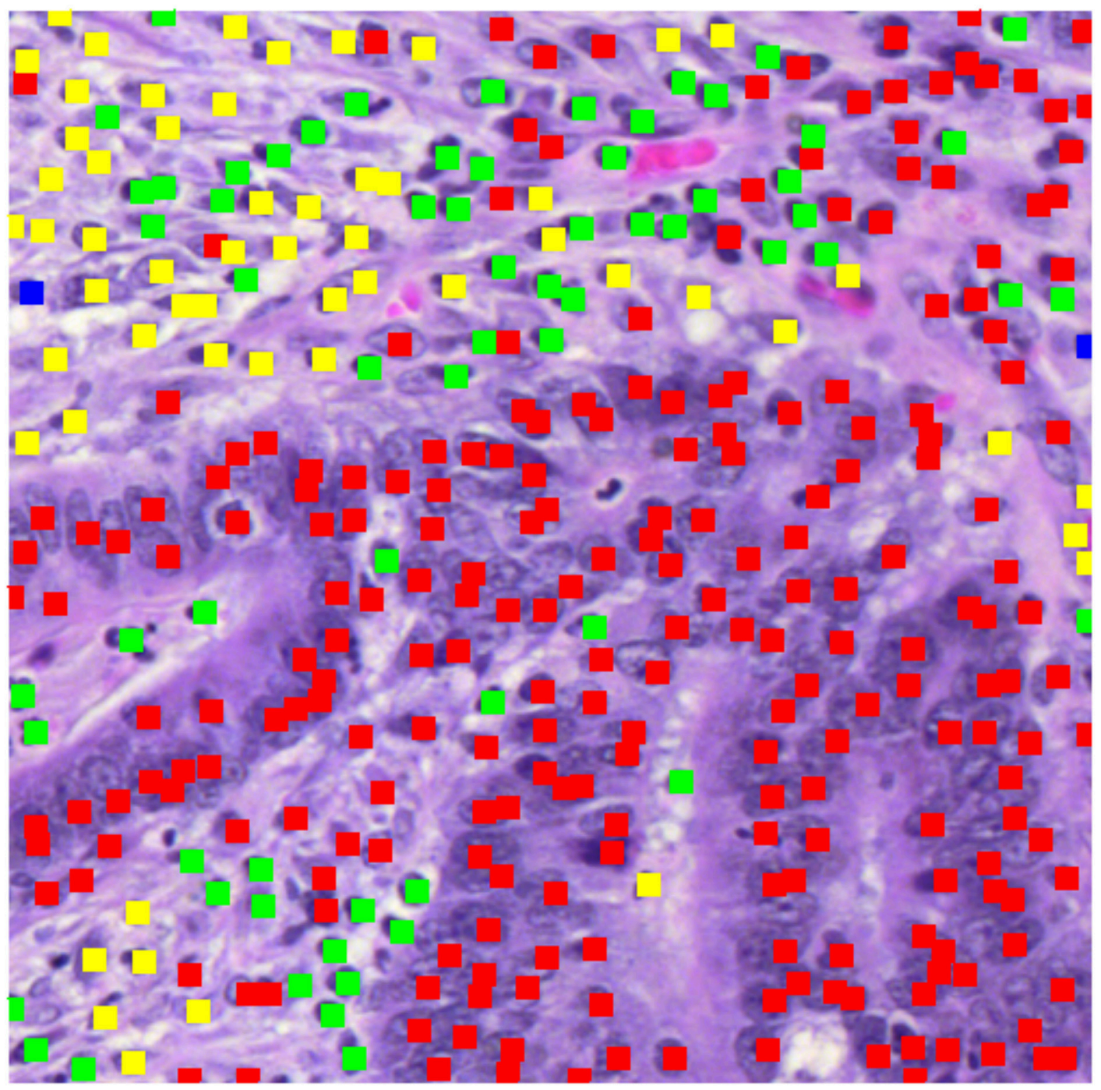
Patch showing types of cells identified.

Figure 2 displays a patch marked with the results of the cell identification algorithm (500 pixels square at 20X). The algorithm has identified a mixture of epithelial cells (red dots) and inflammatory cells (green dots), plus cells identified as fibroblasts (yellow dots). To compute the morphological profile of the patch, we simply count the numbers of different types of cell.

### Training the Cell Identification Algorithm

Training data consisted of 853 hand-marked images, most of which were from the same WSIs described in Sirinukunwattana et al. (2016). The detection algorithm was trained using the method proposed in that publication and described there. The code was implemented in Matconvnet (Vedaldi and Lenc, 2015). The same clustering algorithm as detailed in Sirinukunwattana et al. (2016) was applied to the probability map output by the convolutional neural network and generated the locations of cell nuclei.

The classification model, based on the Tensorflow “cifar10” model (Krizhevsky, 2009), was trained with Tensorflow (Abadi et al., 2016), using the Pycharm IDE. The layers of the classification CNN were the same as those defined in the “cifar10” model (Tensorflow, 2018), and the following hyperparameters were applied: (batch size = 128, moving average decay = 0.9999, number of epochs per decay = 350, learning rate decay factor = 0.1, initial learning rate = 0.1, maximum number of steps = 1,000,000).

The training data used in classification was marked with the locations of different types of cells. The data set included the 100 training images described in Sirinukunwattana et al. (2016) plus patches from the same WSIs and new ones. Sub-patches for training the classification network were generated by selecting 51 × 51 pixel images around hand-marking points. There were 111,659 of these, from which smaller patches of size 33 × 33 pixels were extracted subject to random displacements that allowed for inaccuracies in location (an average of up to ±5 pixels) and each which was augmented in training by four extra images generated by rotation and reflection. All processing was done at 20X (0.5 microns/pixel). The average RGB intensities of the training patches were recorded for later use in standardization. An accuracy of 84% was achieved in evaluation of classification using a hold-out set.

### Experimental Dataset

Colorectal cancer data from The Cancer Genome Atlas (TCGA) has yielded a molecular characterization of human colon and rectal data (Cancer Genome Atlas Network, 2012). With a view to the creation of image profiles, diagnostic images of colon cancer (from haemotoxilyn and eosin formalin embedded samples) were downloaded from the TCGA COAD data set, via the Genomic Data Commons Portal (2018)^1^ COAD, the TCGA colon cancer dataset, contains 400+ diagnostic images, stored in SVS format, most of which have a resolution of 40X (0.25 microns/pixel). Of the COAD set 142 images were selected from a single site, the “AA” site.

### Implementation of Profile Generation

#### Segmentation

Each WSI was segmented into foreground (tissue present) and background (no tissue present) regions using an entropy-based algorithm which created a foreground mask (See Figure 1B). The WSI was tiled with square patches 500 pixels in width (20X). Patches that overlapped with the foreground mask were denoted as foreground patches.

#### Random Sampling—Cell Identification

In the case of RS and for each experimental run, n_T_ patches were randomly sampled from the set of n_F_ foreground patches. The cell detection algorithm was applied to each patch individually. The detection component calculated the haemotoxylin channel and supplied it to the detection CNN. The classification module extracted small patches around each detected point, normalized them collectively, using the average intensities saved from the training stage, and applied the classification algorithm to each patch individually, generating a set of patch types which were used to calculate morphological profiles.

Denoting the profile of patch *t* by *f*_*tij*_, where each WSI is labeled with index *i* the whole-slide profile *fw*_*ij*_ is:

(3) $$\begin{array}{r} fw_{ij}=\frac{\sum_{t=1}^{n_{T}}f_{tij}}{n_{T}}\#\left(3\right) \end{array}$$

### Systematic Random Sampling—Cell Identification

The following version of SRS was implemented. As with RS a sample size was specified: in this case a nominal sample size n_NOM_. A coarse tiling of the WSI used *sample grids*, squares that each contained g × g patches. With SRS one patch was sampled randomly from each sample grid. If the tile was a foreground tile, then the cell identification algorithm was applied to the patch and the resulting profile was added to a list of profiles associated with the WSI. Otherwise, if the patch was a background patch, it was ignored. The whole-slide profile was calculated by averaging the profiles in the list. The choice of *g*, the dimension of the sample grid, depended on the number of foreground tiles in the image as well as on the nominal sample size. The size of the sample grid is a function of three quantities, n_NOM_, the minimum number of tiles to be sampled; γ, the fraction of tiles in the image that are not artifacts; and n_F_ the number of foreground tiles,

(4) $$\begin{array}{r} g=floor\left(\sqrt{\frac{\gamma n_{F}}{n_{NOM}}}\right)\#\left(4\right) \end{array}$$

**Figure 3:**
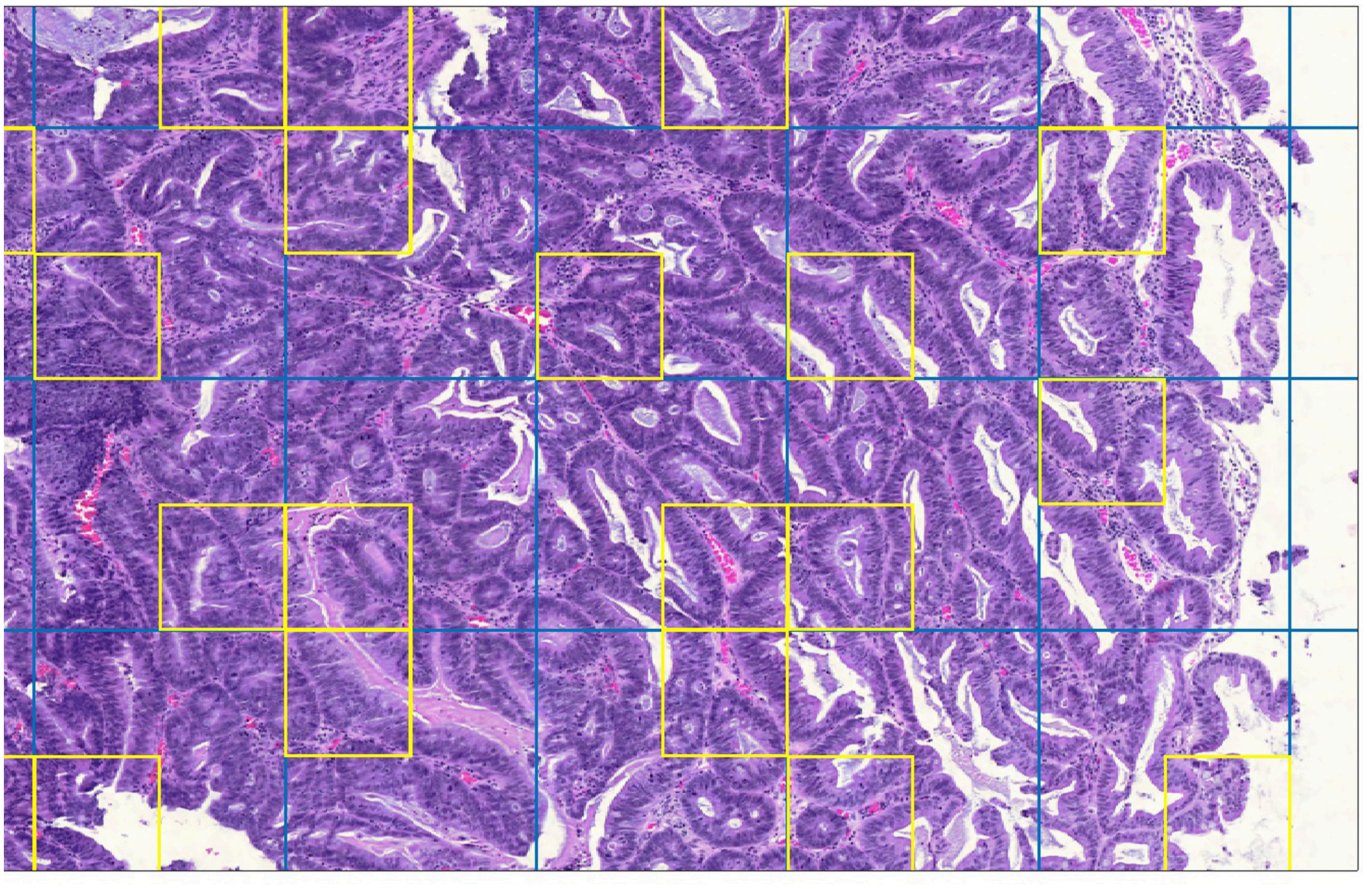
Detail of whole-slide image showing sample grids and selected patches.

Figure 3 is a detail of a whole slide split into sample grids with divisions indicated by blue lines. Each sample grid contains 3 × 3 tiles. The tiles selected by SRS for processing by cell identification are outlined in yellow. Not every sample grid in the figure contains a yellow square: if the sample grid contains only background tiles no tile will be selected; or if a background tile happens to be selected then it is not processed.

Note that a straightforward gray-detection algorithm was used to identify patches containing artifacts. The percentage of patches containing artifacts γ was estimated by sampling patches.

### Evaluation of Profile Generation

In five of the TCGA diagnostic images 1,500 cells were hand-marked by a pathologist. Cells were classified as normal epithelial cells, malignant epithelial cells, inflammatory cells or as fibroblasts. Patches containing hand-marked cells were run through the cell identification algorithm, and the accuracies of detection and classification were computed. (Note that the two types of epithelial cells were merged into one, because the cell identification algorithm did not distinguish them). Both detection and classification achieved 65% accuracy on average (Table 1).

**Table 1:** Detection and classification accuracy.

**Patient ID**	**Detection accuracy**	**Classification accuracy**
AA-3543	0.85	0.66
AA-3845	0.68	0.76
AA-3864	0.62	0.81
AA-3986	0.61	0.90
AA-A02J	0.50	0.66
Average	0.65	0.76

### Experiments With Sampling

Both sampling policies, RS and SRS, were applied using the following nominal sample sizes: 25, 50, 100. For both RS and SRS and for each nominal sample size two batch runs were executed. In each batch run the sampling policy was applied to the 142 whole slide images. The batch runs of RS were done after those for SRS using the actual sample sizes generated by SRS, ensuring that the runs could be compared for accuracy.

**Figure 4:**
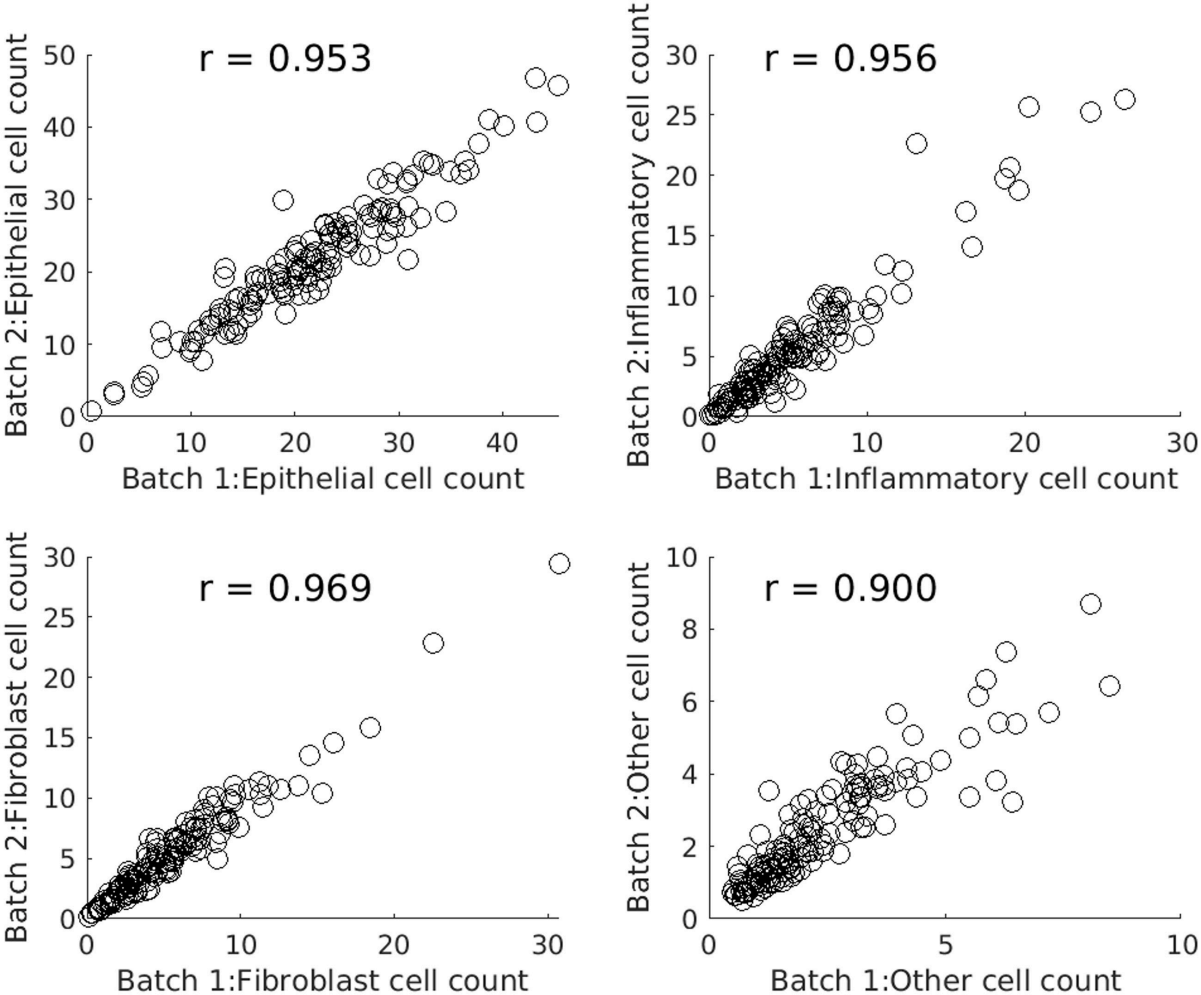
Scatterplots comparing batch runs of SRS (systematic random sampling).

Figure 4 comprises four scatterplots. Each scatterplot displays results for one type of cell and each point on a scatterplot corresponds to one of the images from the experimental dataset. X values are profile features calculated in the first batch run and Y values are the corresponding features output by the second batch run.

Table 2 compares SRS and RS for a range of sample sizes and cell types. Table entries have been computed as follows. For a given nominal sample size, cell type, and WSI, the difference between the features output by two batch runs is calculated. The absolute difference is taken, averaged over all 142 images, and divided by the global average for that cell type to create a *relative batch difference*. As would be expected, the relative batch difference decreases with increasing sample size. SRS performs better than RS in all cases. For example, for epithelial cells, SRS average relative batch differences are fractions 0.80, 0.74, and 0.60 of the corresponding RS values.

**Table 2:** Comparison of RS and SRS.

**Sample size (Nominal number of tiles)**	**25**	**50**	**100**
Epithelial cells	*Global average (per 100 micron square):* 21.3 cells
RS–relative batch diff.	11.30%	8.10%	5.80%
SRS–relative batch diff.	9.20%	6.00%	3.50%
Inflammatory cells	*Global average:* 5.52 cells
RS–relative batch diff.	19.20%	12.50%	8.30%
SRS–relative batch diff.	17.40%	7.60%	6.50%
Fibroblasts	*Global average:* 5.64 cells
RS–relative batch diff.	14.50%	11.00%	8.00%
SRS–relative batch diff.	13.80%	8.10%	4.80%
“Other” cells	*Global average:* 2.43 cells
RS–relative batch diff.	24.30%	16.90%	9.90%
SRS–relative batch diff.	20.10%	11.50%	7.80%

### Correlation Matrix

Table 3 shows the correlations between counts of the four types of cells. A-priori one might expect cell counts to be negatively correlated because cells are competing for space in the tissue. Of the six correlations in the lower left of the matrix four are negative: however, the number of fibroblasts is positively related to the number of “Other” cells. Possibly, the algorithm is misidentifying cells here, or there may be a genuine biological connection.

**Table 3:** Correlation matrix of cell counts.

	**Epithelial**	**Inflammatory**	**Fibroblast**	**Other**
Epithelial	1			
Inflammatory	0.20	1		
Fibroblast	−0.59	−0.34	1	
Other	−0.63	−0.13	0.56	1
	Epithelial	Inflammatory	Fibroblast	Other

### Morphological Profiles and Clinical Variables

Preprocessing of the clinical data associated with the 142 images in the data set identified 14 clinical variables of interest. (Variables with large numbers of missing values were excluded, as were variables with constant values). Each variable was cross-tabulated against each of the four profile features, or correlation coefficients were calculated, or a MANOVA was performed. Where the clinical variable was a binary categorical variable, *t-*tests were used to compare the mean value of the profile variable by clinical group. For example, metastasis was grouped by value as “M0” or “M1” and it was natural to compare the average numbers of different types of cells in the two groups.

Table 4 shows the six clinical variables for which the (uncorrected) *t*-test had a *p* ≤ 0.05 for at least one of the four cell types. The other clinical variables were also tested, but no significant relationships were observed and we do not show these results. Table 4 shows the name of the clinical variable in the first column followed by the categories of interest and the number of patients in each category. In lines containing cell types, the average value of the cell count is shown for each category, followed by the *p*-value. The significance value of 0.05, appropriate to a single test has been adjusted using the Benjamini-Hochberg correction (Hochberg and Benjamini, 1990; Benjamini and Hochberg, 1995) and is shown in the column labeled “BH *p*-value.”

**Table 4:** Associations between cells counts and clinical variables.

**Clinical variable**			***p*-value**	**BH *p*-value**	**BH sig**
**Metastasis**	**M0 (*****n*** **=** **120)**	**M1 (*****n*** **=** **21)**			
*Epithelial*	22.1	17.2	0.00152	0.0411	Y
*Inflammatory*	5.8	4.0	0.0372	0.0411	Y
*Fibroblast*	5.3	7.7	0.0156	0.0429	Y
*Other*	2.1	3.5	0.00506	0.0482	Y
**Residual tumor**	**R0 (*****n*** **=** **117)**	**R2 (*****n*** **=** **20)**			
*Epithelial*	22.1	17.7	0.0130	0.0438	Y
*Inflammatory*	5.8	4.4	0.0506	0.0393	**N**
*Fibroblast*	5.3	7.8	0.0179	0.0420	Y
*Other*	2.2	3.3	0.0100	0.0464	Y
**Vascular invasion**	**NO (*****n*** **=** **64)**	**YES (*****n*** **=** **73)**			
*Fibroblast*	4.6	6.4	0.00661	0.0473	y
**Venous invasion**	**NO (*****n*** **=** **98)**	**YES (*****n*** **=** **30)**			
*Epithelial*	22.9	19.4	0.0111	0.0455	Y
*Fibroblast*	4.8	6.4	0.0116	0.0446	Y
**Mucinous carcinoma**	**NO (*****n*** **=** **120)**	**YES (*****n*** **=** **20)**			
*Inflammatory*	5.9	3.4	0.00361	0.0491	Y
**Vital status**	**Alive (*****n*** **=** **130)**	**Dead (*****n*** **=** **12)**			
*Inflammatory*	5.70	3.80	0.0488	0.0402	**N**

Differences between the two categories for *metastasis* had significant *p*-values for all cell types. Compared with M0 (colorectal cancer without evidence of distant metastasis), the category M1, where metastasis was present, had increased numbers of fibroblasts and “Other” cells and fewer epithelial cells and inflammatory cells. The presence of *residual tumor* was also associated with more fibroblasts and “Other” cells and fewer epithelial cells and inflammatory cells. Both *vascular invasion* and *venous invasion* were associated with increased numbers of fibroblasts. Venous invasion was associated with fewer epithelial cells.

Mucinous carcinomas were associated with fewer inflammatory cells than were non-mucinous carcinomas. Finally, the 12 patients who were recorded as dead when added to the TCGA repository were also likely to have fewer inflammatory cells detected than patients who were recorded as alive, although the associated *p*-values were not significant.

Note that the remaining clinical variables, for which no associations were found, were as follows: Gender, Age, T Stage, N Stage, History of colon polyps, History of other malignancy, Anatomic neoplasm subdivision (Tumor Location—left side vs. right side), and CEA level.

## Discussion

### Conclusions

In this application statistical sampling of patches from whole-slides images proved to be worthwhile: significant improvements in performance were achieved with very little loss of accuracy. Systematic random sampling was markedly more accurate than straightforward random sampling. For example, with a sample size of 100, and considering epithelial cell counts the batch difference indicator was 3.5% for systematic random sampling and 5.8% for basic random sampling (Table 3 above).

The profiles being computed were particularly suitable for random sampling because the features of interest were *additive* over regions in the images. In applications where the regions of interest are sparse and spatially concentrated, adaptive sampling may be more appropriate. The two examples from the literature, discussed in the introduction use random sampling to find regions of interest followed by adaptive sampling to narrow the search.

Statistically significant associations between morphology and various clinical variables were found in this study. The TNM grading system used in cancer treatment considers tumor penetration, nodes, and metastasis (National Cancer Institute, 2018). Of these three indicators significant associations were found for metastasis, for all four types of cell.

There were five clinical variables for which we found significant relationships with morphological features. Four clinical variables had significant associations with fibroblast counts: in each case higher fibroblast counts were associated with poorer values of the clinical variable. This is not unexpected (Hewitt et al., 1993). In a review of the role of cancer-associated fibroblasts in the tumor microenvironment, Kalluri (2016) refers to fibroblasts as the “cockroaches” of the human body and states that they play an important role in tumorigenesis and cancer progression.

Two clinical variables were associated with differences in inflammatory cell counts, namely metastasis, and mucinous carcinoma. Poor values of the clinical variables were associated with lower numbers of inflammatory cells, which might be expected, in the light of the positive role of tumor infiltrating lymphocytes in slowing down disease progression (Nosho et al., 2010; Nigro et al., 2016).

Finally, metastasis, residual tumor, and venous invasion were related to lower numbers of epithelial cells.

The morphological features extracted from the 142 diagnostic images from the COAD data set may be regarded as expressions of *cellularity*. Cellularity is a familiar concept in pathology: here each morphological feature corresponds to the spatial density of the corresponding cell type.

### Future Directions

In addition to the cellularity features studied here, other features may be calculated using deep learning. Such features include *tumor budding* which is the presence of single tumor cells or small clusters of up to five cells in the stroma and which is associated with aggressive cancer (Ueno et al., 2002; De Smedt et al., 2016; Koelzer et al., 2016). In addition, (Konishi, 2018)suggest that *poorly differentiated clusters, perineural invasion, and desmoplastic reaction* are also important in diagnosis. Another morphology of interest is that of *serrated cancers* in which the colonic glands are of distinctly serrated form (García-Solano et al., 2012; Murcia et al., 2016).

Jass (2007) classified colorectal cancers according to molecular features, observing that they are related to morphological features such as the number of tumor infiltrating lymphocytes, differentiation, presence of dirty necrosis, serration, tumor budding, mucinous/not mucinous, and presence of an expanding invasive margin. Felipe De Sousa et al. (2013) reported that serrated cancers have distinct molecular features. Deep learning has recently been used to predict diagnostic molecular features from morphology, e.g., for lung cancer (Coudray et al., 2018), and breast cancer (Couture et al., 2018). It is to be expected that future work with deep learning will enable morphological, clinical and molecular data to be linked.

### Extending the Analysis

We have shown experimentally that a cell identification algorithm using deep learning can uncover interesting relationships between tissue morphology and a range of clinical variables and that systematic sampling of tissue regions can improve performance without losing accuracy.

The experimental results in this paper were obtained from a single TCGA site. The analysis should be extended to all sites in the TCGA colon cancer repository. In the experiments carried out here, standardization was straightforward, using the pooled average intensities of a group of WSIs to normalize data. Unfortunately, there is no guarantee that this approach will always be successful. Standardization techniques that cater for the many different originating sites in TCGA should be used. Carried out effectively, standardization ensures that reproducible morphological features are generated.

## Data Availability

Publicly available datasets were analyzed in this study. This data can be found here: https://gdc.cancer.gov.

## Author Contributions

MS was responsible for aspects of sampling, handled the data, developed the code and did the numerical analysis, and wrote the report. KH did hand-marking of the TCGA histology slides and provided advice on both histology and clinical data. NR contributed the general framework for the deep learning approach and provided both general specific guidance.

### Conflict of Interest Statement

The authors declare that the research was conducted in the absence of any commercial or financial relationships that could be construed as a potential conflict of interest.

## Footnotes

^1^https://gdc.cancer.gov
